# Association of promoter methylation with histologic type and pleural indentation in non-small cell lung cancer (NSCLC)

**DOI:** 10.1186/1746-1596-6-48

**Published:** 2011-06-04

**Authors:** Meiju Ji, Yong Zhang, Bingyin Shi, Peng Hou

**Affiliations:** 1 Department of Endocrinology, The First Affiliated Hospital of Xi'an Jiaotong University College of Medicine, Xi'an 710061, the People's Republic of China; 2 Department of Pathology, The First Affiliated Hospital of China Medical University, Shenyang 110001, the People's Republic of China

## Abstract

**Background:**

Lung cancer is a major cause of death worldwide. Gene promoter methylation is a major inactivation mechanism of tumor-related genes, some of which can be served as a biomarker for early diagnosis and prognosis evaluation of lung cancer.

**Methods:**

We determined the promoter methylation of 6 genes using quantitative methylation-specific PCR (Q-MSP) technique in 96 clinically well-characterized non-small cell lung cancer (NSCLC).

**Results:**

Highly frequent promoter methylation was found in NSCLC. With 100% diagnostic specificity, high sensitivity, ranging from 44.9 to 84.1%, was found for each of the 6 genes. Our data also showed that promoter methylation was closely associated with histologic type. Most of genes were more frequently methylated in squamous cell carcinomas (SCC) compared to adenocarcinomas (ADC). Moreover, promoter methylation significantly increased the risk of pleural indentation in NSCLC.

**Conclusion:**

Our findings provided evidences that multiple genes were aberrantly methylated in lung tumorigenesis, and demonstrated the promoter methylation was closely associated with clinicopathologic characteristics of NSCLC. More importantly, we first revealed promoter methylation may be served as a potentially increased risk factor for pleural indentation of NSCLC patients.

## Background

Lung cancer is a leading cause of cancer death worldwide, accounting for 30% of all cancer-related deaths [1]. The 5-year survival rate between 1996-2004 is 16%-significantly lower than that of other major cancers [2]. Owing to the rapid industrialization and increase in the smoking consumption in society, lung cancer presents as the number one cancer type of threats in China [3]. Epidemiological evidence has documented that approximately 41.8 men and 19.3 women per 100,000 Chinese individuals died of lung cancer in 2005 [4]. Lung cancer is generally classified into two major histological categories, small cell lung cancer (SCLC) and non-small cell lung cancer (NSCLC). The latter accounts for approximately 85% of lung cancer [2]. Approximately 25-33% of NSCLC patients present with stage I or II disease, which permits surgical resection with curative intent. However, despite surgery, approximately 30-40% of patients with NSCLC who have discrete lesions and histologically negative lymph nodes die of recurrent disease [5]. Despite the fact that the cause of most lung cancer is well know, the disease has proven difficult to diagnosis early and treat successfully, reflecting limited advances in our understanding of the molecular mechanisms underlying lung carcinogenesis and individual susceptibility to lung cancer.

In addition to genetic factors [6-8], epigenetic alterations play an important role in lung cancer development and result in changes in gene function [9]. DNA methylation is an epigenetic event whose pattern is altered frequently in a wide variety of human cancers, including promoter-specific hypermethylation as well as genome-wide hypomethylation [10,11]. Gene promoter hypermethylation is among the earliest and most common alterations in human cancers, including NSCLC, which leads to gene silencing and inactivation [12,13].

In the present study, we sought to identify DNA methylation profiles in NSCLC and their association with known or suspected cancer risk factors. For this, we used quantitative methylation-specific PCR (Q-MSP) to evaluate promoter methylation of a panel of cancer-associated genes in a large cohort of clinically well-characterized NSCLC samples, including calcitonin-related polypeptide alpha (*CALCA*), E-cadherin (*CDH1*), death-associated protein kinase 1 (*DAPK1*), iroquois homeobox 1 (*IRX2*), TIMP metallopeptidase inhibitor 3 (*TIMP3*), and paired box 6 (*PAX6*). These genes were potentially important in NSCLC, some of which have been assessed by others but some of which have not been evaluated previously.

## Methods

### Study subjects and DNA isolation

Ninety-six tumor samples and 15 nonmalignant lung samples were obtained from NSCLC patients who underwent curative resection at the First Affiliated Hospital of China Medical University, with approval by the institutional review board of the Hospital. None of these patients received chemotherapy and radiotherapy before the surgery. Informed consent was obtained from each patient before the surgery. All of the samples were histologically examined by a pathologist at Department of Pathology of the Hospital to identify the type and other clinical characteristics of the tumors. The clinical files of these patients are shown in Table 1. Samples were prepared and genomic DNA was isolated from paraffin-embedded samples as previously described [14]. Briefly, after a treatment for overnight at room temperature with xylene to remove pareffin, tissues were digested with 1% sodium dodecyl sulfate (SDS) and 0.5 mg/ml proteinase K at 48°C for 48 h, with addition of several spiking aliquots of concentrated proteinase K to faciliate digestion. DNA was subsequently isolated using standard phenol/chloroform protocol, and was dissolved in distilled water and stored at -80°C until use.

**Table 1:** Clinicopathologic characteristics of NSCLC cases

Characteristics	No. of patients (%)
Gender	

Male	66 (69)

Female	30 (31)

Age (mean years ± S.D.)	58.9 ± 9.2

≤ 60	56 (58)

> 60	40 (42)

Smoking history (pack-years)	

0	30 (31)

1-39	35 (37)

≥ 40	31 (32)

Tumor size (mean cm ± S.D.)	3.9 ± 1.7

1-3	37 (38)

3-5	44 (46)

> 5	15 (16)

Histologic type	

Adenocarcinoma (ADC)	30 (31)

Brochoalveplar cell (BAC)	6 (6)

Adenocarcinoma (non-BAC)	24 (25)

Squamous (SCC)	66 (69)

Histologic stage	

I	54 (56)

II	32 (34)

III	10 (10)

Lymph node metastasis	

No	70 (73)

Yes	26 (27)

Pleural indentation	

No	75 (78)

Yes	21 (22)

Invasion or Adhesion	

No	65 (68)

Yes	31 (32)

### Sodium bisulfite treatment

DNA from the primary tumors and nonmalignant lung samples was subjected to bisulfite treatment as described previously [14]. Briefly, a final volume of 20 μl of H_2_O containing 1-2 μg genomic DNA, 10 μg salmon sperm DNA, and 0.3M NaOH was incubated at 50°C for 20 min to denature the DNA. The mixture was then incubated for 2-3 h at 70°C in 500 μl of a freshly prepared solution containing 3 M sodium bisulfite (Sigma, Saint Louis, MO), 10 mM hydroquinone (Sigma, Saint Louis, MO). Subsequently, the DNA was recovered by a Wizard DNA Clean-Up System (Promega Corp., Madison, WI) following the instructions of the manufacturer, followed by ethanol precipitation, and resuspension in 30 μl of deionized H_2_O. After bisulfite processing, all unmethylated cytosine residues converted to uracil, whereas the methylated cytosine residues remained unchanged. Bisulfited-modified DNA samples were stored at -80°C until use.

### Quantitative methylation-specific PCR (Q-MSP) analysis

After sodium bisulfite conversion, the methylation analysis was performed by the fluorescence-based quantitative PCR assay as described previously [14]. Briefly, the Q-MSP amplification was carried out in triplicate for each samples in a final reaction mixture of 20 μl containing 3 μl bisulfite-treated DNA, 600 nM each primer, 200 nM TaqMan probe, 5.5 mM MgCl2, 1 U platinum *Taq *polymerase, 200 μM each of deoxyguanosine triphosphate, and 2% Rox reference. After an initial denaturation step at 95°C for 2 min, 40 cylces of 15 sec at 95°C and 60 sec at 60°C for annealing and extension were run using an ABI 7500 Fast Real-Time PCR System (Foster City, CA). Normal leukocyte DNA was methylated *in vitro *with Sss I methylase (New Engliand Biolabs, Beverly, MA) to generate completely methylated DNA as a positive control. Each plate contained triplicate samples and multiple water blanks, as well as serial dilutions of positive methylated control to construct the standard curve. The internal reference gene *β-actin *was used to normalize the amount of input DNA. The primers and TaqMan probes used in the present study were presented in Table 2. The relative degree of methylation of each sample was calculated using the method described previously [15].

**Table 2:** Quantitative methylation-specific PCR primer and TaqMan probe sequences used in the present study

Genes	Forward primer sequence (5'→3')	Probe sequence (5'→3')	Reverse primer sequence (5'→3')
***CALCA***	GTTTTGGAAGTATGAGGGTGACG	6FAM-ATTCCGCCAATACACAACAACCAATAAACG-TAMRA	TTCCCGCCGCTATAAATCG

***CDH1***	AATTTTAGGTTAGAGGGTTATCGCGT	6FAM-CGCCCACCCGACCTCGCAT-TAMRA	TCCCCAAAACGAAACTAACGAC

***DAPK1***	GGATAGTCGGATCGAGTTAACGTC	6FAM-TTCGGTAATTCGTAGCGGTAGGGTTTGG-TAMRA	CCCTCCCAAACGCCGA

***IRX2***	GCGGGTCGTTTAGGTTAGTATTCG	6FAM-CCCTCCATCCACGCCCGACCGAAA-TAMRA	CGCCGAACAACGAACAAAACG

***TIMP3***	GCGTCGGAGGTTAAGGTTGTT	6FAM-AACTCGCTCGCCCGCCGAA-TAMRA	CTCTCCAAAATTACCGTACGCG

***PAX6***	ATATAGGACGGCGGTTTAGGTTG	6FAM-CCCAAAATCCGACCGACTCCAACCCCTA-TAMRA	TTCCGACCGAACGAAAACCTAC

***β-Actin***	TGGTGATGGAGGAGGTTTAGTAAGT	6FAM-ACCACCACCCAACACACAATAACAAACACA-TAMRA	AACCAATAAAACCTACTCCTCCCTTAA

### Statistical analysis

Promoter methylation was considered present if the ratio was above a certain cut-off value. The relative level of methylation varied significantly among the 6 genes and therefore cut-off points were studied for each gene individually. To set up cut-off value for each gene for detection of NSCLC, we used the Medcalc Software (MedCalc Software bvba, Belgium) to construct receiver operating characteristic (ROC) curves. The area under the curve of ROC curve is a measure of the ability of a continuous marker to accurately classify tumor and non-tumor tissue. Such a curve is a plot of sensitivity *vs*. 1 minus specificity values associated with all dichotomous markers that can be formed by varying the value threshold (or cut-off value) used to designate a marker "positive". Factors associated with patients and tumor characteristics were assessed univariately with chi-square tests for trend and logistic regression. In the final analysis, multivariable adjustments were made to adjust for the potentially confounding effects of smoking history, histologic type, lymph node metastasis, and pleural indentation. *P *< 0.05 was considered to be statistically significant. All statistical analyses were performed using the SPSS statistical package (11.5, Chicago, IL, USA).

## Results

### Demographics

In the present study, we chose genes which were potentially important in NSCLC and determined the promoter methylation of these 6 genes using Q-MSP assay in 96 well-characterized NSCLC patients. As shown in Table 1 the mean age of the 96 NSCLC cases was 58.9 years. Males were more than females (69% *vs*. 31%). Sixty-nine percent of patients reported a history of smoking, with 32% reporting at least 40 life-time pack-years of smoking. Ninety percent of NSCLC cases had surgical stage I or II disease and 84% had tumors < 5 cm. By histology, 31% of tumors were adenocarcinomas (ADC), including 6% bronchioloalveolar carcinomas (BAC) and 25% non-BAC adenocarcinomas, and 69% were squamous cell carcinomas (SCC). The cases with lymph node metastasis, pleural indentation and invasion or adhesion were in 26/96 (27%), 21/96 (22%), and 31/96 (32%), respectively.

### Frequent promoter methylation in NSCLC

**Figure 1:**
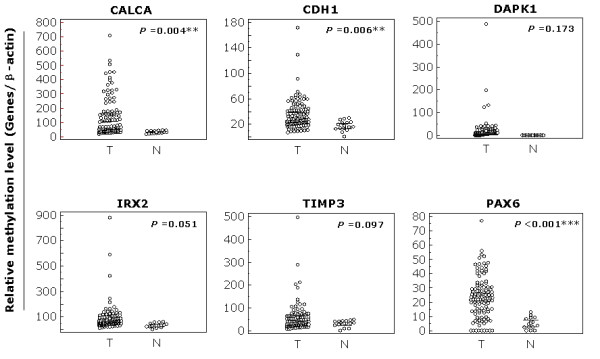
**Distribution patterns of promoter methylation of the 6 genes in NSCLC**. Q-MSP was performed as described in Materials and Methods. The relative methylation level (on *Y *axis) is represented by ratios of candidate gene/*β-actin *× 1000, except for *PAX6*/*β-actin *× 100. Horizonal lines indicate a 95% normal confidence interval for the sample mean. T, tumor tissues; N, nonmalignant lung tissues. **, *P *< 0.01; ***, *P *< 0.001.

**Figure 2:**
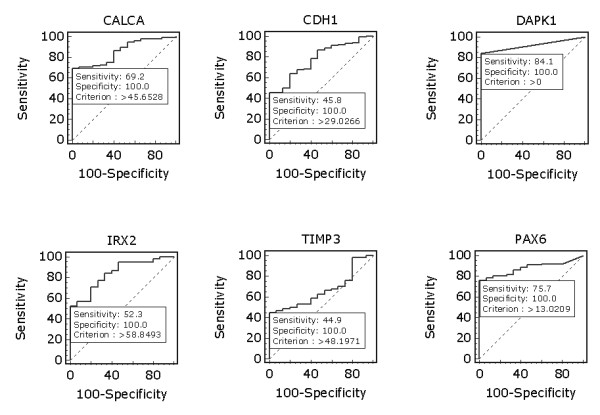
**Receiver operating characteristic (ROC) curves for the 6 genes in NSCLC**. All tumor and nonmalignant lung tissues for which there was complete DNA methylation data were used for the analyses. The ROC curves plot sensitivity *vs*. 100-specificity. The determined cut-off values for *CALCA, CDH1, DAPK1, IRX2, TIMP3*, and *PAX6 *were 45.7, 29.0, 0, 58.8, 48.2, and 13.0, respectively.

We examined promoter methylation of *CALCA, CDH1, DAPK1, IRX2, TIMP3*, and *PAX6 *using Q-MSP in a cohort of 96 NSCLC. As shown in Figure 1, the overall methylation level of each gene was higher in tumor tissues than in nonmalignant lung tissues. Among the 6 genes examined, statistical significances were observed in *CALCA *(*P *< 0.01), *CDH1 *(*P *< 0.01), and *PAX6 *(*P *< 0.001). We set up appropriate cut-off values to distinguish NSCLC from nonmalignant lung tissues, and determine diagnostic sensitivity and specificity. As shown in Figure 2, with 100% diagnostic specificity for each of the 6 genes, the sensitivity of *CALCA, CDH1, DAPK1, IRX2, TIMP3*, and *PAX6 *was 69.2%, 45.8%, 84.1%, 52.3%, 44.9%, and 75.7%, respectively.

### Association of promoter methylaton with clinicopathological charactristics of NSCLC

Among all clinicopathologic characteristics, methylation levels varied substantially by histologic type (Table 3). Methylation level of most of genes was higher in SCC than in ADC, particularly in *CDH1 *gene (*P *< 0.05) (Table 3). There was a significant difference in the methylation level of *DAPK1 *gene between lymph node metastasis group and nonmetastasis group (40.3 ± 100.5 in the former *vs*. 12.3 ± 18.5 in the latter, *P *< 0.05) (Table 3). The methylation level of *CALCA *gene was significant higher in the patients with invasion or adhesion compared with patients without invasion or adhesion (174.5 ± 160 *vs*. 115.1 ± 122.5, *P *< 0.05) (Table 3). The methylation level of *CALCA *gene was found to be significantly associated with smoking history (155.2 ± 150.2 in smokers *vs*. 89.6 ± 92.7 in non-smokers, *P *< 0.05) (Table 3). Our data showed that the methylation level of most of genes was not associated with quantity of cigarette smoking (Pack-years) (Additional file 1). Of note, there was a significant difference in the methylation level of *PAX6 *gene between patients with age > 60 years and ≤ 60 years (28.6 ± 16.2 in the former *vs*. 20.2 ± 12.4 in the latter, *P *< 0.05). In addition, the methylation level of *CALCA *gene was also found to be significantly associated with tumor stage (217.7 ± 186.7 in patients with histoligical satge ≥ III *vs*. 125 ± 129 in patients with histological stage < III, *P *< 0.05) (Additional file 1). The methylation level of several genes was associated with increased tumor size, including *CALCA, DAPK1, IRX2*, and *TIMP3 *genes, although these associations did not reach statistical difference (Additional file 1).

**Table 3:** Association of the level of promoter methylation with clinicopathologic charactristics of NSCLC

Genes	Gender (n = 96)			Histologic type (n = 96)			Smoking history (n = 96)		
			
	Female	Male	*P*	SCC	ADC	*P*	Y	N	***P***
						
	(n = 30)	(n = 66)		(n = 66)	(n = 30)		(n = 66)	(n = 30)	
***CALCA***	145.0 ± 149.6	112.1 ± 106.5	0.28	152.1 ± 152.1	96.4 ± 90.6	0.07	155.2 ± 150.2	89.6 ± 92.7	0.03*

***CDH1***	33.1 ± 18.7	31.5 ± 21.2	0.71	35.4 ± 19.2	26.3 ± 18.6	0.03*	32.2 ± 18.7	33.4 ± 21.3	0.77

***DAPK1***	18.5 ± 59.6	23 ± 45.2	0.71	26.2 ± 65.8	5.9 ± 7.6	0.09	17.4 ± 59.6	25.4 ± 45.2	0.52

***IRX2***	96.4 ± 131.5	63.9 ± 34.8	0.19	92.3 ± 115.7	72.9 ± 103.6	0.43	95.3 ± 132.1	66.3 ± 36.1	0.24

***TIMP3***	60.5 ± 72.8	50.9 ± 39.1	0.50	64.9 ± 71.7	41.3 ± 39.8	0.09	61.5 ± 72.8	48.8 ± 38.9	0.37

***PAX6***	23.4 ± 15.1	24.2 ± 13.8	0.78	24.0 ± 15.5	23.1 ± 12.8	0.79	23.9 ± 15.6	23.3 ± 12.6	0.85

**Genes**	**Lymph node metastasis (n = 96)**			**Pleural indentation (n = 96)**			**Invasion or Adhesion (n = 96)**		
			
	**Y**	**N**	***P***	**Y**	**N**	***P***	**Y**	**N**	***P***
						
	**(n = 26)**	**(n = 70)**		**(n = 21)**	**(n = 75)**		**(n = 31)**	**(n = 65)**	

***CALCA***	175.7 ± 175.1	119.5 ± 119.1	0.08	129.9 ± 132.5	136 ± 140.2	0.86	174.5 ± 160	115.1 ± 122.5	0.048*

***CDH1***	31.0 ± 13.9	33.1 ± 21.2	0.64	35.3 ± 21.2	31.8 ± 18.9	0.47	30.7 ± 16.6	33.5 ± 20.7	0.52

***DAPK1***	40.3 ± 100.5	12.3 ± 18.5	0.03*	12.4 ± 11.9	21.9 ± 62.3	0.49	10.9 ± 10.6	24.2 ± 66.6	0.27

***IRX2***	42.7 ± 51	91.2 ± 127.2	0.47	73.1 ± 38.4	89.9 ± 124.9	0.54	71.8 ± 48.3	93.1 ± 131.6	0.38

***TIMP3***	69.3 ± 91	53.2 ± 51	0.28	53.9 ± 42.9	58.6 ± 69.2	0.77	52.7 ± 53.1	59.8 ± 69.1	0.61

***PAX6***	23.2 ± 14.2	23.9 ± 14.9	0.83	25.2 ± 13.9	23.3 ± 14.9	0.59	25.8 ± 15.7	22.7 ± 14.1	0.32

In univariate analyses, the promoter methylation of most of genes was more frequent in SCC compared to ADC, particularly in *CDH1*(OR = 2.63, 95% CI = 1.05-6.60), *DAPK1 *(OR = 6.64, 95% CI = 1.85-23.8), *IRX2 *(OR = 2.89, 95% CI = 1.17-7.13), and *TIMP3 *(OR = 2.51, 95% CI = 1.01-6.21) (Table 4). In addition, methylation of *CALCA *(OR = 2.13, 95% CI = 1.01-4.47) and *TIMP3 *(OR = 1.88, 95% CI = 1.01-3.49) was significantly associated with histologic stage (Table 4). However, there was not significant association between promoter methylation and other clinocopathologic characteristics, including gender, age, smoking history, quantity of cigarette smoking, tumor size, lymph node metastasis, pleural indentation, and invasion or adhesion (Table 4). In order to assess the independent associations between promoter methylation and smoking history, histologic type, lymph node metastasis, and pleural indentation, we conducted multiple multivariable logistic regressions (Table 5). In multivariable analyses adjusting for potential false discovery rate associated with multiple comparisons (6 different models), methylation of most of genes ramained associated with histologic type. The promoter methylation of *CDH1*(OR = 6.77, 95% CI = 1.71-26.8), *DAPK1 *(OR = 10.7, 95% CI = 2.16-52.6), *IRX2 *(OR = 6.38, 95% CI = 1.17-23.0), and *TIMP3 *(OR = 3.37, 95% CI = 1.03-11.0) genes was significant more likely in SCC compared to ADC (Table 5). However, after adjustment for smoking history, histologic type, and lymph node metastasis, the promoter methylaton of each gene was more frequent in patients with pleural indentation compared with without pleural indentation (Table 5), particularly in *CDH1*(OR = 5.27, 95% CI = 1.27-21.9), *DAPK1 *(OR = 6.98, 95% CI = 1.06-45.8), and *IRX2 *(OR = 3.89, 95% CI = 1.01-15.0) genes, suggesting that promoter methylation of these genes may be a potential risk of pleural indentation in NSCLC.

**Table 4:** Promoter methylation in NSCLC-univariate associations with clinicopathologic characteristics (OR^† ^and 95% CI)

Genes	Male vs. Female	Age (per 10 years)	Smoking history	Pack-years^1^	SCC *vs*. ADC
***CALCA***	0.73 (0.28-1.90)	0.94 (0.58-1.52)	1.43 (0.57-3.57)	0.88 (0.51-1.51)	2.21 (0.89-5.47)

***CDH1***	2.13 (0.86-5.22)	0.83 (0.53-1.30)	1.41 (0.59-3.39)	0.91 (0.55-1.50)	2.63 (1.05-6.60)*

***DAPK1***	1.45 (0.43-4.87)	1.16 (0.60-2.24)	2.11 (0.64-6.92)	1.83 (0.84-3.98)	6.64 (1.85-23.8)*

***IRX2***	1.29 (0.54-3.06)	0.76 (0.48-1.19)	1.06 (0.45-2.52)	0.91 (0.55-1.50)	2.89 (1.17-7.13)*

***TIMP3***	1.09 (0.46-2.60)	1.09 (0.70-1.71)	1.09 (0.46-2.60)	1.27 (0.76-2.11)	2.51 (1.01-6.21)*

***PAX6***	1.04 (0.37-2.88)	1.19 (0.70-2.03)	0.78 (0.27-2.25)	0.93 (0.51-1.69)	1.04 (0.37-2.88)

**Genes**	**Tumor size^2^**	**Lymph node metastasis**	**Pleural indentation**	**Invasion or Adhesion**	**Histologic stage^3^**

***CALCA***	1.01 (0.55-1.88)	1.74 (0.62-4.91)	1.18 (0.41-3.41)	2.44 (0.88-6.79)	2.13 (1.01-4.47)*

***CDH1***	1.20 (0.67-2.13)	1.02 (0.41-2.51)	1.79 (0.67-4.76)	0.96 (0.41-2.27)	1.11 (0.62-2.02)

***DAPK1***	1.81 (0.72-4.54)	5.17 (0.64-41.95)	1.63 (0.33-8.02)	1.70 (0.43-6.67)	1.24 (0.50-3.08)

***IRX2***	1.57 (0.88-2.83)	1.17 (0.47-2.88)	1.37 (0.52-3.63)	0.86 (0.36-2.01)	1.14 (0.63-2.07)

***TIMP3***	1.67 (0.92-3.01)	2.05 (0.82-5.10)	1.16 (0.44-3.05)	0.69 (0.29-1.66)	1.88 (1.01-3.49)*

***PAX6***	0.78 (0.40-1.54)	0.74 (0.26-2.09)	2.04 (0.54-7.69)	1.84 (0.61-5.56)	1.76 (0.79-3.93)

† OR: odds ratio with 95% confidence interval.

1 Pack-years (0; > 0 and < 40; ≥ 40).

2 Tumor size (> 1 and ≤3; > 3 and ≤5; > 5).

3 Histologic stage (I; II; III).

* Significant at *P *< 0.05.

**Table 5:** Promoter methylation in NSCLC-multivariable models assessing smoking history, histologic type, lymph node metastasis, and pleural indentation (OR^† ^and 95% CI)

Genes	Smoking history	SCC *vs*. ADC	Lymph node metastasis	Pleural indentation
***CALCA***	1.07 (0.40-2.88)	2.66 (0.85-8.32)	1.43 (0.46-4.41)	2.19 (0.61-7.79)

***CDH1***	0.98 (0.37-2.58)	6.77 (1.71-26.8)*	0.78 (0.29-2.13)	5.27 (1.27-21.9)*

***DAPK1***	0.83 (0.19-3.73)	10.7 (2.16-52.6)*	3.07 (0.32-29.4)	6.98 (1.06-45.8)*

***IRX2***	0.69 (0.26-1.83)	6.38 (1.77-23.0)*	0.85 (0.31-2.33)	3.89 (1.01-15.0)*

***TIMP3***	0.78 (0.30-2.02)	3.37 (1.03-11.0)*	1.75 (0.65-4.75)	2.66 (0.77-9.16)

***PAX6***	0.67 (0.21-2.08)	1.82 (0.50-6.57)	0.77 (0.25-2.38)	2.66 (0.58-12.3)

† OR: odds ratio with 95% confidence interval, adjusted for multiple comparisions.

* Significant at *P *< 0.05.

## Discussion

We assessed the promoter methylation of the 6 genes in a large cohort of well-characterized NSCLC subjects using Q-MSP technique in the present study, including *CALCA, CDH1, DAPK1, IRX2, TIMP3*, and *PAX6. CALCA *is known to encode a peptide hormone that plays a role in maintenance of calcium levels in blood serum and T-and B-cell regulation in certain malignancies, which is frequently methylated in multiple types of cancer [16,17]. *CDH1 *is a classical cadherin from the cadherin superfamily, which encodes a calcium dependent cell-cell adhesion protein. Epigenetic inactivation of *CDH1 *is thought to contribute to progression in cancer by increasing proliferation, invasion, and/or metastasis [18]. *DAPK1 *encodes a structurally unique calcium/calmodulin-dependent serine/threonine kinase which acts as a positive regulator of apoptosis. It is frequently methylated in human cancers as a tumor suppressor gene [19]. IRX2 is a member of the Iroquois homeobox transcription factor family, which is involved in developmental pattern formation in multiple organs such as the brain and heart [20]. It is highly specific for tumor-associated methylation, and little or no methylation is found in nonmalignant lung tissue [21]. TIMP3 is an angiogenesis inhibitor, and its epigenetic inactivation is associated with neovascularization and invasion in human malignancy [22]. PAX6, a transcription factor, has currently been suggested to function as a tumor suppressor in glioblastoma and to act as an early differentitation marker for neuroendocrine cells [23], which is frequently methylated in human cancers [21,24]. Similarly, our findings also showed that 3 of 6 genes had significantly higher methylation level in tumor tissues than nonmalignant lung tissues, including *CALCA, CDH1*, and *PAX6 *genes. Importantly, with 100% diagnostic specificity, excellent sensitivity, ranging from 44.9 to 84.1%, was found for each of the 6 genes. The high specificity and frequency of these methylation markers make them excellent candidates for future applications developed for early diagnosis and prognosis evaluation of lung cancer.

Given smoking plays the central role in lung cancer development, it is somewhat surprising that we did not find significant association between promoter methylation of most genes and smoking history, in agreement with most studies [25-28]. However, several previous studies have reported aberrant DNA methylation of tumor-related genes was associated with tobacco smoking [29-32]. It is possible that lung cancer as a result of tobacco smoking is a complex disease with many unique genetic and epigenetic features. Better understanding of the molecular mechanisms underlying this disease would undoubtedly improve the outcomes of patients with smoking-associated lung cancer.

Our findings of substantial differences in promoter methylation depending on histologic typing of NSCLC have been reported to some degree in the literatures. Unlike what observed in the present study, a number of others have noted that adenomatous polyposis coli (*APC*), cyclin D2 (*CCND2*), potassium voltage-gated channel, subfamily H (eag-related), member 5 (*KCNH5*), and runt-related transcription factor 2 (*RUNX2*) genes were significantly more frequently in ADC compared to SCC [33-36]. Similar to the present study, although no statistical significance was observed, promoter methylation of *DAPK1 *gene was detected with higher frequency in SCC compared to ADC [36]. Conversely, there was not significant difference in promoter methylation of *CDH1 *gene between SCC and ADC in this literature [36]. Anyway, we believe that our findings that promoter methylation of several genes is more frequent in SCC compared to ADC, particularly *IRX2 *gene, are new to the literature. Moreover, methylation level of *DAPK1 *and *CALCA *genes was significantly associated with lymph node metastasis and invasion/adhesion, suggesting that methylation degree of these genes might contribute to oncologic outcomes of NSCLC patients.

A very recent study showed that a number of important differences in frequency of promoter methylation in females compared to males, suggesting that promoter methylation is associated with gender [36]. However, these substantial differences have not been consistently noted in the previous literatures [35,37-39]. Similarly, promoter methylation was also not significantly associated with gender in the present study. It is possible that the differences in the promoter methylation associated with gender may be related to the geographical or cultural differences in carcinogen exposures, including cigarette smoking, dietary factors, occupational and environmental chemical exposure [40-42]. The discrepant results might also have been due to genetic differences of the study populations. A previous study indicated that patients harboring functional polymorphic variants of glutathione S-transferase pi 1 (*GSTP1*) had a higher risk of promoter hypermethylation of O-6-methylguanine-DNA methyltransferase (*MGMT*) gene [43], suggesting that functional variants in the genes involved in the folate metabolism, DNA methylation, carcinogen metabolosim and the repair of methylation may play an important role in the susceptibility to promoter methylation. However, it is possible that these differences are attributable to chance as a result of the relatively small number of the study subjects examined. Therefore, further study with more subjects will be needed.

Interestingly, multivariable analyses revealed that the promoter methylation of a number of genes had signficant higher frequency in patients with pleural indentation compared with without pleural indentation, suggesting that promoter methylation may be a potentially increased risk for pleural indentation of NSCLC patients. Pleural indentation is a well-known imaging sign on chest computed tomography (CT) that suggests a possible pleural invasion by peripheral NSCLC, particularly ADC [44,45]. A previous study showed that pleural involvement was significantly correlated with a poor prognosis in NSCLC, suggesting that pleural involvement may be one of most important factors to affect on the prognosis of NSCLC [46]. Although the degree of pleural invasion is clinically important, the accurate preoperative evaluation is sometimes difficult. CT and magnetic resonance imaging (MRI) are usually used in the evaluation of tumor extent, and diagnosis of chest wall invasion, pleural dissemination and pleural effusion can be easily made preooperatively by this way [47]. However, pleural dissemination and/or pleural effusion, which were not documented preoperatively, were sometimes revealed during operation. In addition, the accruate visceral pleural invasion can not be made with preoperative CT or MRI. In the present study, our findings suggested that promoter methylation of certain genes can increase the risk of pleural indentation in NSCLC. DNA methylation is the earliest and most frequent molecular events in human tumorigenesis. Detection of aberrant methylation using some high-senstive approaches can be thus used to predict and evaluate the pleural invovlement in NSCLC.

## Conclusion

In summary, in the present study, we found a panel of methylated genes that differentiate tumor tissues from nonmalignant lung tissues, which was strongly associated with clinicopathologic characteristics of NSCLC. Importantly, our data first revealed that promoter methylation may be a potentially increased risk factor for pleural indentation of NSCLC patients.

## Authors' contributions

MJ and PH conceived and designed the experiments. MJ and YZ performed the experiments. MJ and PH collected the samples and analyzed the data. BS and PH contributed reagents/materials/analysis tools. PH Wrote the paper. All authors are in agreement with the content of the manuscript and this submission.

## Competing interests

The authors declare that they have no competing interests.

## Supplementary Material

Additional file 1 **Table S1**. Association of the level of promoter methylation with age, pack-years, tumor size and histoligic stage in NSCLC Click here for file
