# Supplementary material for: Association of promoter methylation with histologic type and pleural indentation in non-small cell lung cancer (NSCLC)
Source: Diagn Pathol. 2011 Jun 4;6:48. doi: 10.1186/1746-1596-6-48 (PMC3123260; doi:10.1186/1746-1596-6-48)
Supplement: Additional file 1 — Table S1. Association of the level of promoter methylation with age, pack-years, tumor size and histoligic stage in NSCLC [file 1746-1596-6-48-S1.DOC]

**Additional file 1**. Association of the level of promoter methylation with age, pack-years,

tumor size and histoligic stage in NSCLC

| **Genes** | **Age (n =96)** | | |  | **Pack-years (n =96)** | | |
| --- | --- | --- | --- | --- | --- | --- | --- |
|  | > 60 | ≤ 60 | *P* |  | > 30 | ≤ 30 | *P* |
|  | (n =40) | (n =56) |  |  | (n =34) | (n =62) |  |
| ***CALCA*** | 140.6 ± 128.1 | 130.5 ± 145.4 | 0.73 |  | 150.5 ± 145.8 | 126 ± 133.7 | 0.41 |
| ***CDH1*** | 32.5 ± 23.9 | 32.6 ± 15.7 | 0.97 |  | 27.9 ± 13.5 | 35.1 ± 21.7 | 0.08 |
| ***DAPK1*** | 14.3 ± 22 | 23.9 ± 70.1 | 0.41 |  | 22.2 ± 82.4 | 18.6 ± 33.1 | 0.76 |
| ***IRX2*** | 72.7 ± 68.8 | 95.9 ± 134.3 | 0.32 |  | 83.8 ± 142.4 | 87.6 ± 92.2 | 0.87 |
| ***TIMP3*** | 53.4 ±42.3 | 60.5 ± 76.3 | 0.59 |  | 67.5 ± 90.5 | 52.1 ± 43.7 | 0.26 |
| ***PAX6*** | 28.6 ± 16.2 | 20.2 ± 12.4 | 0.005* |  | 25 ± 16.9 | 23 ± 13.3 | 0.53 |
| **Genes** | **Tumor size (n =96)** | | |  | **Histologic stage (n =96)** | | |
|  | >4 | ≤ 4 | *P* |  | ≥Ⅲ | < Ⅲ | *P* |
|  | (n =32) | (n =64) |  |  | (n =10) | (n =86) |  |
| ***CALCA*** | 171 ± 182.6 | 116.6 ± 106 | 0.07 |  | 217.7 ± 186.7 | 125 ± 129 | 0.04* |
| ***CDH1*** | 31.8 ± 13.9 | 32.9 ± 21.7 | 0.79 |  | 33.7 ± 13.3 | 32.4 ± 20 | 0.84 |
| ***DAPK1*** | 28.7 ± 82.2 | 15.5 ± 30.1 | 0.27 |  | 10.2 ± 6.6 | 21 ± 58.4 | 0.56 |
| ***IRX2*** | 100.2 ± 150.1 | 79.2 ± 87.4 | 0.39 |  | 83.6 ± 62.4 | 86.5 ± 116.5 | 0.94 |
| ***TIMP3*** | 72.9 ± 92.2 | 49.9 ± 43.1 | 0.09 |  | 67 ± 80 | 56.4 ± 62.6 | 0.62 |
| ***PAX6*** | 24.5 ± 16.4 | 23.3 ± 13.8 | 0.69 |  | 25.8 ± 9.7 | 23.4 ± 15.1 | 0.64 |

* Significant at *P* <0.05.
